# Prevalence, risk factors and impact of severe burnout syndrome in 12 Uruguayan ICUs

**DOI:** 10.1186/cc11098

**Published:** 2012-03-20

**Authors:** G Burghi, J LAmbert, M Chaize, C Quiroga, G Pittini, M Cancela, H Bagnulo, S Chevret, E Azoulay

**Affiliations:** 1 Hospital Maciel, Montevideo, Uruguay; 2 Saint Louis Hospital, Paris, France; 3 Hospital Español, Montevideo, Uruguay; 4 CAAMEPA, Pando, Uruguay; 5 Hospital de Clínicas, Montevideo, Uruguay

## Introduction

Burnout syndrome (BOS) is defined as a state of emotional fatigue that leads to a loss of motivation, usually progressing towards feelings of inadequacy and failure. Severe BOS is relevant as it leads to loss of psychological well-being, increased absenteeism and turnover, and deterioration in the quality of care provided to patients. The objective was to determine the prevalence of BOS among Uruguayan ICU clinicians. To evaluate personal or organization characteristics associated with the development of severe BOS.

## Methods

A survey was conducted in 12 Uruguayan adult ICUs. The level of BOS was evaluated on the basis of the Maslach Burnout Inventory (MBI score). ICU, patient, and clinician characteristics were assessed for their association with the prevalence of severe BOS (that is, highest MBI scores). All variables with *P *< 0.2 in univariate analysis were included in a model of ordinal regression. *P *< 0.05 was considered statistically significant.

## Results

A total of 364 questionnaires were evaluated, including 282 nurses and 82 ICU physicians. The prevalence of severe BOS was 51% among ICU physicians and 42% in nursing staff. For ICU nurses, factors independently associated with lower MBI scores were the following: work on fixed days (OR 0.6; 95% CI 0.3 to 0.9; *P *= 0.01), integrated in-ICU working groups (OR 0.6; 95% CI 0.3 to 0.9; *P *= 0.02), good relationships with physicians (OR 0.8; 95% CI 0.7 to 0.9; *P *= 0.008) and good relationships with supervisors (OR 0.8; 95% CI 0.7 to 1; *P *= 0.05). In contrast, at least one death over the last week was associated with higher MBI score (OR 2; 95% CI 1.2 to 3.2; *P *= 0.008). For ICU physicians, not being partnered was independently associated with higher MBI scores. Conversely, good relationships with colleagues was associated with lower MBI scores (OR 0,5; 95% CI 0.3 to 0.8; *P *= 0.004). Interestingly, this study confirms that clinicians with severe BOS had increased burden such as sleep disorders, libido troubles, lack of memory, inadequate money management as well as the wish to leave the ICU.

## Conclusion

The prevalence of severe BOS is very high among ICU workers in Uruguay. We have identified different risk factors associated with the development of severe BOS. These results confirm previous findings and highlight that strategies to decrease BOS in ICU clinicians are urgently warranted.

